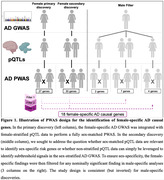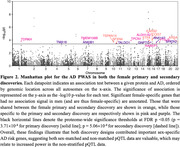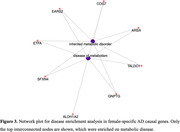# Sex‐Specific Causal Genes for Alzheimer’s Disease via Proteome‐Wide Association Studies

**DOI:** 10.1002/alz.095188

**Published:** 2025-01-09

**Authors:** Danielle M. Reid, Michael E. Belloy

**Affiliations:** ^1^ Washington University in Saint Louis, Saint Louis, MO USA

## Abstract

**Background:**

Women are at higher risk of developing Alzheimer’s disease (AD). Sex differences in AD pathobiology and prevalence appear to be partially attributed to genetics, but sex‐stratified genome‐wide association studies (GWAS) of AD have demonstrated a relative paucity of associated variants, which may reflect challenges of limited power. Furthermore, these studies lack information regarding the impact on sex‐specific proteins and pathways relevant to AD, which limits clinical translation and targeted drug development. Sex‐stratified protein quantitative trait locus (pQTL) studies pose to address these limitations. We hypothesized that directly integrating brain pQTL data with the largest to date sex‐stratified AD GWAS would increase power to reveal sex‐specific AD associated genes.

**Method:**

Sex‐stratified GWAS of AD from Belloy et al. 2023 included 410,276 female and 385,813 male non‐Hispanic White individuals of European ancestry. Human brain proteome data with matching genome‐wide data were available from Wingo et al. 2022 (N_Subjects_ = 722) and 2023 (N_Women_ = 507; N_Men_ = 301). Briefly, genetic variants were associated with proteins through the FUSION package to estimate effects on protein abundance and produce non‐sex‐stratified (N_Proteins_ = 2,934) and sex‐stratified protein‐specific variant weights (N_Proteins_ = 1,800 for males; N_Proteins_ = 2,463 for females). This approach retains only proteins that show significant genetic associations (i.e., protein levels displaying significant heritability). Proteome‐wide association studies (PWAS) were performed via FUSION combining sex‐stratified AD GWAS with non‐sex‐stratified and sex‐stratified protein weights (**Figure‐1**). Enrichment/pathway analysis for gene and disease ontology was conducted via the DOSE package (R 4.3.3).

**Result:**

Sex‐matched AD PWAS identified 27 and 2 AD causal genes for females and males, respectively. Sex‐stratified AD GWAS integrating non‐stratified protein weights identified 30 causal genes in females and 7 in males. Upon filtering results of both discoveries for sex specificity, 18 female and 2 male AD causal genes were prioritized (**Figure‐2**). Enrichment/pathway analysis of female‐specific AD causal genes highlighted metabolic‐related health outcomes (**Figure‐3**).

**Conclusion:**

We performed sex‐stratified PWAS of AD and identified 18 female‐specific AD causal genes, of which 8 strikingly converged onto metabolic disorders as a putative female‐specific AD pathway. These findings increase our understanding of AD pathogenesis and may inform therapeutic target development relevant to sex‐specific precision medicine. Supporting analyses in additional proteogenomic resources are ongoing.